# Public opinion on policy interventions for regulating four unhealthy commodity industries: a cross-sectional online survey of a representative sample of British adults 2023

**DOI:** 10.1186/s12889-025-25271-x

**Published:** 2025-11-21

**Authors:** Emily Reed, Vera Buss, Corrina Bebbington, Laura Bunce, Hazel Cheeseman, Katherine Severi, Katharine Jenner, Jeff Collin

**Affiliations:** 1 Action on Smoking and Health, Unit 2.9, The Foundry, 17 Oval Way, London, SE11 5RR United Kingdom; 2 University College London, Gower Street, London, WC1E 6BT United Kingdom; 3 SPECTRUM Consortium, Usher Institute, The University of Edinburgh, 5-7 Little France Road, Edinburgh BioQuarter – Gate 3, Edinburgh, EH16 4UX United Kingdom; 4 Institute of Alcohol Studies, Canopi, 82 Tanner Street, , London, SE1 3GN United Kingdom; 5 Obesity Health Alliance, London, United Kingdom; 6 Global Health Policy Unit, School of Social & Political Science, University of Edinburgh, 15a George Square, Edinburgh, EH8 9LD United Kingdom

**Keywords:** Public health policy, Alcohol, Tobacco, Unhealthy food and drink, Gambling, Industry

## Abstract

**Background:**

Tobacco, alcohol, unhealthy food and drink, and gambling are all unhealthy commodity industries which have profound public health consequences. The tactics of these industries intending to undermine policies and scientific evidence are often similar. To counter this, greater coherence in regulatory response is needed within, and across, policy spheres. Understanding public opinion is key in this context.

**Objectives:**

This study assessed public support for policies targeting unhealthy commodities and factors associated with support, including sociodemographic features, current smoking, excess alcohol consumption, obesity and daily gambling.

**Design:**

Across-sectional design, where anonymous data was drawn from the annually commissioned Action on Smoking and Health Smokefree British survey conducted online between 22^nd^ February and 15^th^ March 2023.

**Participants:**

12,271 British adults, invited to participate from the online YouGov panel. Weighted by sociodemographic characteristics to be representative of British adults.

**Outcome measures:**

Percentage support for policies to reduce harm from unhealthy commodities; and associations between the level of support, sociodemographic features, and exposure to unhealthy commodities.

**Analysis:**

Missing data were imputed using multiple imputations and data were weighted to match the British population. The degree of public support for policies were descriptively assessed and associations between this support, sociodemographic characteristics and the harmful consumption of unhealthy commodities, were measured using logistic regression.

**Main findings:**

Respondents strongly supported measures targeting industry interactions with government, and the majority believed that public health policy should be protected from industry influence (69.8% for alcohol, 75.6% for gambling, 75.0% for tobacco and 68.2% for unhealthy food and drinks manufacturers). There was also majority support for industry levies (77.1% for tobacco, 74.2% for gambling, 61.9% for alcohol, 59.5% for unhealthy food and drink), however, taxation and advertising bans received more mixed responses. Some sociodemographic factors (age and social grade) and exposure to some unhealthy commodities (current smoking, alcohol intake >14 units per week, daily gambling) were associated with differential levels of support for public health policies. However, sex and obesity did not have meaningful associations.

**Conclusion:**

The public are generally supportive of public health policies to reduce harm from tobacco, alcohol, gambling and unhealthy food and drink.

**Supplementary Information:**

The online version contains supplementary material available at 10.1186/s12889-025-25271-x.

## Summary box

### What is already known on this topic

Despite similarities between industry tactics in promoting tobacco, alcohol, gambling and unhealthy food and drink, policy approaches to tackling these unhealthy commodities are highly variable.

### What this study adds

Our study shows that the public are generally supportive of measures to tackle tobacco, alcohol, gambling and unhealthy food and drink, with stronger support for measures tackling tobacco and gambling. The public are particularly supportive of interventions with a clear focus on industries. Respondents were more likely to favour fiscal policies directly impacting on an industry (levies), compared with those that increased prices for consumers. There was broad support for measures to protect policy-making from industry influence. These cross-industry findings are significant in considering scope to develop cohesive approaches to tackling unhealthy commodity industries.

## Background

Alcohol, tobacco, unhealthy food and drink, and gambling have profound public health consequences. Common to all these unhealthy commodities is that their health harms and social impacts are substantially driven by the actions, and deliberative tactics, of industries that profit from their consumption [1]. The tactics that these unhealthy commodity industries use to target people, and especially vulnerable populations, are well recognised and broadly include: lobbying and other activities to influence political process; manufacturing doubt and shifting blame; aggressive marketing and advertising; deploying corporate social responsibility and partnerships; and pushing for self-regulation [1, 2]. Such industry actions negatively shape the conditions in which people are born, grow, live, work, and age; and there has been increasing recognition of the commercial determinants of health as key social determinants in recent years [1, 3]. Consequently, the products of unhealthy commodity industries are associated with significant health harms, including links with many non-communicable diseases [1]. There are also wider costs to society, including widening inequality, lost productivity, increasing social care needs, and negative impacts on earning and employment potential [4–7].

There has been longstanding recognition of tobacco industry tactics to undermine policy and science, which has led to increasingly comprehensive action to counteract tobacco industry influence. The UK is a party to the World Health Organisation treaty, the Framework Convention on Tobacco Control, meaning that protection of public health policy from the actions of the tobacco industry has a legal underpinning. This has enabled regulation, taxation and bans, which have led to significant reductions in smoking prevalence over several decades [8]. Tobacco-related deaths have subsequently fallen [9]. However, tobacco remains responsible for over 74,000 deaths per year (15% of all deaths in England) [9], and the societal costs of tobacco in England still stand at an estimated £43.7 billion per year [4]. This suggests that even more stringent regulation is required, including that proposed in the upcoming Tobacco and Vapes Bill [10].

Despite increasing recognition in academic and public health communities that the tactics of alcohol, gambling and unhealthy food and drink industries mirror those of Big Tobacco [11, 12]; governments and health system partners have not approached these unhealthy commodity industries in the same way, and continue to develop public-private partnerships with these industries [13]. The policy levers to tackle the harms caused by these commodities are also similar (such as those targeting availability, affordability and promotion of the commodities), but policy approaches have remained siloed by industry and risk factor [14, 15], and progress has been insufficient relative to the health and societal harms from these commodities [16–18].

This is demonstrated with respect to unhealthy food and drink, where companies and industry representatives often continue to be engaged as partners or stakeholders in the development of policies around diet and obesity [19]. Although there have been some incremental policy successes around unhealthy food and drink in recent years, such as the Soft Drinks Industry Levy, progress around the regulation of advertising, product use and environment has stalled [15]. Stalling policy progress and continued industry influence translates negatively for the public’s health, with overweight and obesity rates continuing to rise [20], and consumption of unhealthy food and drink estimated to cause 13% of all deaths in England [21]. This has wider economic impacts, with the societal costs of obesity and overweight in the UK estimated to stand at £126 billion each year [6].

The alcohol industry is also actively involved in policy [22], and best practice policies to prevent alcohol harm are largely absent in England [15]. Meanwhile, alcohol-related deaths are continuing to rise annually in England (over 22,000 deaths reported in 2023) [20], and the societal costs of alcohol are estimated at £27.4 billion each year [7].

Finally, the gambling industry also continues to be considered a legitimate partner in UK gambling policy. This results in industry-favourable legislation, and industry’s key positioning in education, treatment, and research around gambling [23, 24]. This is despite the latest evidence from the Office for Health Improvement and Disparities suggesting that gambling is responsible for between 117 and 496 suicides per year in England, with a societal cost between £241.1 million and £961.7 million each year [25].

To counter the significant health harms that result from the actions of the tobacco, alcohol, unhealthy food and drink, and gambling industries, there is increasing recognition of the need for a coherent, comprehensive and strategic public health approach to tackle the affordability, availability and acceptability of unhealthy commodities [13, 15]. As part of this approach, more effective regulation is needed within and across policy spheres. In this context, understanding public opinion is particularly significant since strong public support can impact on the development of public policy, including in countering the influence of lobbying from powerful industry interests [26]. The present study, therefore, aimed to answer the following research questions: (1) What was the degree of public support for policy action to regulate the tobacco, alcohol, gambling, and unhealthy food and drink industries among UK adults in 2023? (2) Which sociodemographic characteristics and risk factors associated with experiencing harmful effects of the unhealthy commodities were associated with support for policy interventions?

## Methods

### Study design

This study used a cross-sectional design, with anonymous data drawn from the Action on Smoking and Health (ASH) Smokefree GB survey [27]. The STROBE checklist [28] was used in its reporting, as these standards are applicable for cross-sectional study designs (see supplementary material).

### Sample and recruitment

The ASH Smokefree GB Survey is an annually commissioned survey by ASH and is conducted by YouGov (a market research company) [27]. It explores behaviours and attitudes related to smoking and vaping in adults in Great Britain, and includes questions about cross-risk factors related to alcohol, unhealthy food and drink, and gambling.

The 2023 survey was conducted with adults (aged 18 and over) living in Great Britain, invited from YouGov’s panel, between the 22nd of February and the 15th of March [27]. YouGov employs an active sampling method which dynamically evaluates what surveys are available for a particular panel member and weights the data to ensure the sample is representative of British adults. 12,271 adults responded to the invitation informing them around the survey, and then completed the survey online (see supplementary file for survey questions). YouGov adheres to the code of conduct set out by the Market Research Society [29] and participants receive modest financial incentives for taking part (panel members get on average 50 “points” per survey, with an equivalent value of 50 pence). Further information about YouGov’s methodology is available online [30].

### Outcome measures

#### Sociodemographic characteristics

Anonymised information on age, sex, and socioeconomic position (measured using social grades A-E derived from the British National Readership Survey [31] was provided through YouGov’s existing panel participant information. For inclusion in the logistic regression models, socioeconomic position was dichotomised into more (ABC1) and less socioeconomically advantaged (C2DE).

#### Consumption of unhealthy commodities

Consumption of unhealthy commodities were measured in the survey [27] through self-reported smoking status, gambling frequency and weekly alcohol units. Body mass index (BMI) was measured as a proxy of exposure to unhealthy food and drink and as a major health consequence of these products. Questions on smoking and gambling frequency were mandatory, however, alcohol consumption included the options “don’t know” and “prefer not to say” and questions on height and weight, from which BMI was calculated, included a “prefer not to say” option. For inclusion in the logistic regression models, answer options around.

smoking status (never smoked, formerly smoked, non-daily smoker, daily smoking), gambling frequency (every day, not every day but at least once per month, a few times a year or less, never), weekly alcohol units (none, 1–14, 15–35, 35 + units) and BMI (< 18, 18–24.9.9, 25–29.9.9, ≥ 30.0) were dichotomised into currently smoking (yes/no), daily gambling (yes/no), drinking above guideline recommendations (i.e., more than 14 alcohol units per week; yes/no), being obese (i.e., BMI of 30 or above; yes/no).

#### Support for policy measures

Participants were required to answer all questions around support for a variety of policy measures for alcohol, tobacco, gambling and unhealthy food and drink; selecting from “strongly support”, “tend to support”, “neither support nor oppose”, “tend to oppose”, “strongly oppose”, or “don’t know”. However, a question on an alcohol levy was presented in two different formats: “requiring the alcoholic drinks industry to pay a levy to government for measures to reduce and prevent harm from alcohol” and “requiring the alcoholic drinks manufacturers to pay a levy to government for measures to reduce and prevent harm from alcohol”. These two options were randomly assigned to participants and results pooled for analysis.

### Analysis

The analysis was conducted in RStudio (version 2022.07.2, R version 4.2.1). The number and percent of missingness for each variable is reported in the supplementary material. Answer options “prefer not to say” and “don’t know” were considered missing, except for policy support statements where “don’t know” responses were included in the analysis. Missing values were imputed using multivariate imputation by chained equations (mice package in R) [32–34] with 20 imputations and 20 iterations. Variables related to sociodemographic characteristics and harmful consumption of unhealthy commodities were included in the imputation models. Each data set was analysed individually and then the results were combined according to Rubin’s rules [35] to get pooled estimates and 95% confidence intervals (CIs). All data were weighted by age, sex, region (nine English regions, Scotland and Wales), socio-economic position (grades A-E) and ethnicity to be representative of Great British adults. For the first research question, the degree of public support for different policies was plotted. Graphs were produced in Microsoft^®^ Excel for Mac (version 16.78.3). For the second research question, responses were dichotomised, with “tend to support” and “strongly support” grouped together as “supporting” the policy and “neither support nor oppose”, “tend to oppose”, “strongly oppose” and “don’t know” grouped together as “not supporting” the policy. Adjusted odds ratios (OR_adj_) with corresponding 95% CIs were calculated using logistic regression to measure associations between support for different policies and age, sex, social grade, current smoking, BMI ≥ 30, drinking above guideline recommendations, and daily gambling. The respective other variables were used for adjustment.

### Public and patient involvement statement

Patients and the public were not formally involved in the development of this study.

## Results

### Sample characteristics

The survey included 12,271 respondents from England, Scotland and Wales. Weighted and imputed sample characteristics are shown in Table 1. Information on BMI was missing for 1,912 respondents (15.6%) and information on alcohol units consumed was missing for 898 respondents (7.3%).

**Table 1:** Sociodemographic characteristics of sample population (N_unweighted_=12 271, weighted and imputed)

Characteristic	Estimate (95% CI)
Age, mean	48.6 (48.3 to 48.9)
Female, %	51.5 (50.5 to 52.4)
Social grade, %	
A	10.1 (9.6 to 10.6)
B	12.4 (11.8 to 12.9)
C1	30.8 (30.0 to 31.7)
C2	20.9 (20.1 to 21.7)
D	12.5 (11.8 to 13.1)
E	13.4 (12.8 to 14.0)
BMI, %	
<18	2.4 (2.1 to 2.7)
18.0–24.9.0.9	40.1 (39.2 to 41.0)
25.0–29.9.0.9	32.2 (31.3 to 33.0)
≥ 30.0	25.3 (24.4 to 26.1)
Smoking status, %	
Never smoked	54.1 (53.1 to 55.0)
Formerly smoked	33.2 (32.4 to 34.1)
Non-daily smoking	4.4 (4.0 to 4.8)
Daily smoking	8.3 (7.8 to 8.8)
Gambling, %	
Every day	1.4 (1.2 to 1.6)
Not every day but at least once a month	26.3 (25.5 to 27.1)
A few times a year or less	29.8 (29.0 to 30.7)
Never	42.5 (41.6 to 43.4)
Alcohol consumption (units per week), %	
None	40.2 (39.3 to 41.1)
1–14	43.2 (42.3 to 44.1)
15–35	12.2 (11.6 to 12.8)
35+	4.4 (4.0 to 4.7)

### Support for policy measures

#### Industry interference

**Fig. 1:**
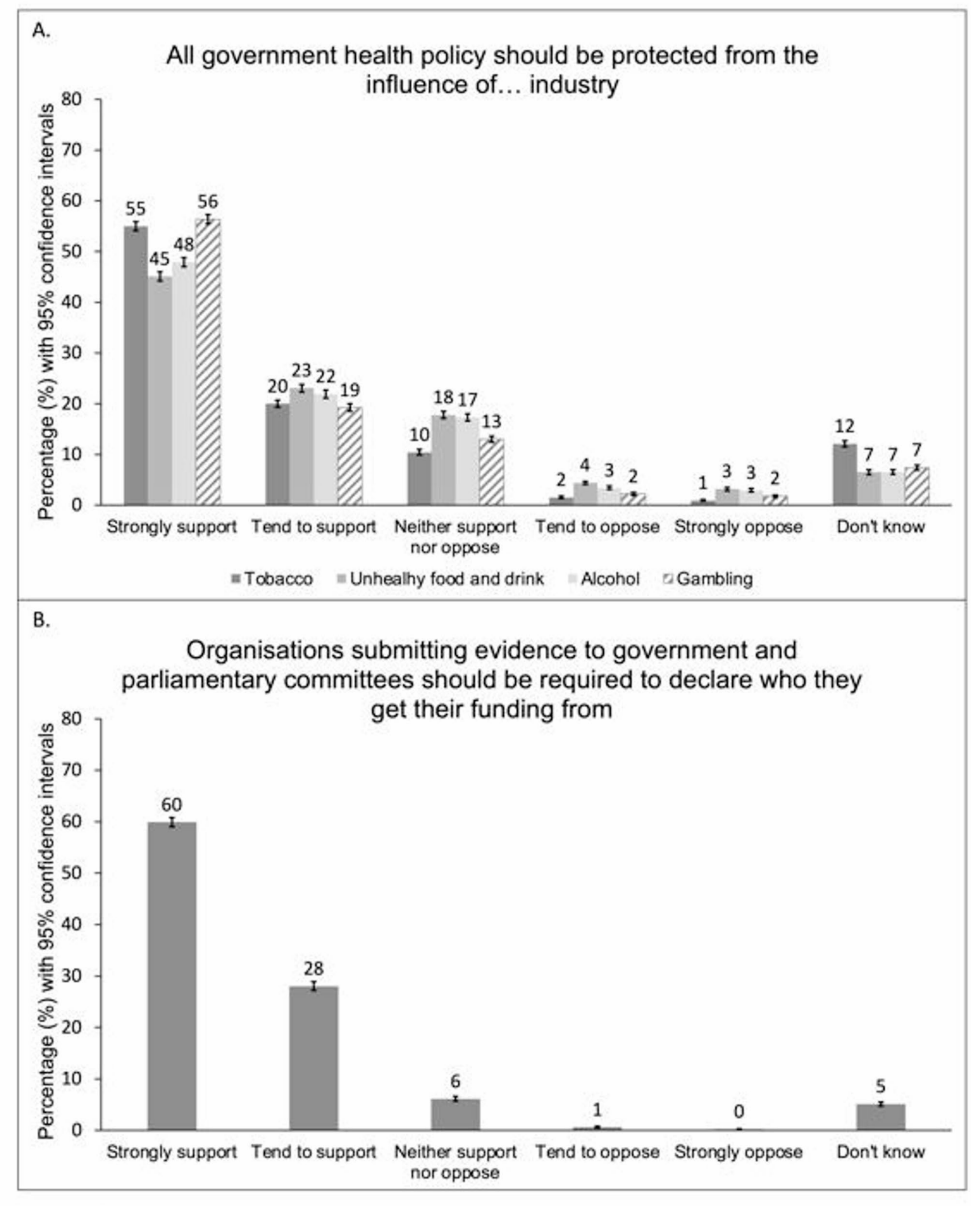
Percentage support for policies targeting industry interactions with government (Weighted, N_unweighted_= 12 271). Legend:(**A**) support for statements: “*All Government health policy should be protected from the influence of [the tobacco industry/unhealthy food and drink manufacturers/the alcohol industry/the gambling industry] and [its/their] representatives.”* (**B**) support for the statement: *“Organisations submitting evidence to government and parliamentary committees should be required to declare who they get their funding from”. The bars in* Fig. 1*represent 95% confidence intervals.*

The survey [27] indicated broad support for measures to restrict industry interference in policymaking. Respondents were supportive of the idea that health policy should be protected from the influence of health-harming industries (Fig. 1A). Support was strongest for protecting health policy from the gambling industry (strongly support 56.4%, 95% CI 55.5% to 57.3%; tend to support 19.2%, 95% CI 18.5% to 20.0%) and the tobacco industry (strongly support 55.0%, 95% CI 54.1% to 55.9%; tend to support 20.0%, 95% CI 19.3% to 20.7%). Similarly, respondents strongly supported the idea that organisations submitting evidence to government or parliamentary committees should need to declare their funding (Fig. 1B), with 87.9% either supporting or strongly supporting this (strongly support 59.9%, 95% CI 59.0% to 60.8%; tend to support 28.0%, 95% CI 27.2% to 28.9%).

#### Fiscal measures

**Fig. 2:**
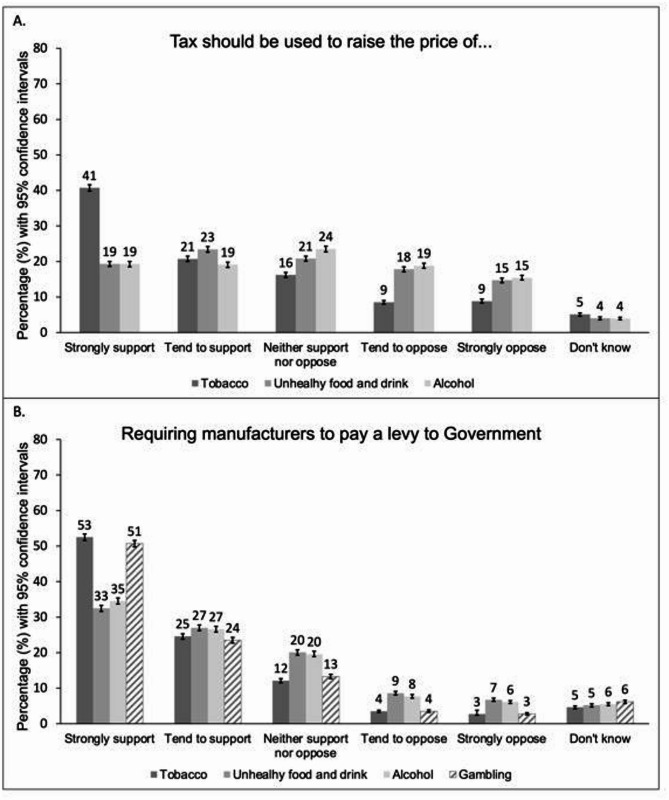
Percentage support for fiscal policies (Weighted, N_unweighted_= 12 271). Legend:(**A**) support for the statements: *“tax should be used to increase the price of tobacco products 5% above the rate of inflation each year/tax should be used to increase the price of [unhealthy food and drink/alcohol]”*. (**B**) support for the statements: *“requiring (tobacco manufacturers/unhealthy food and drink manufacturers/the alcohol industry/the gambling industry) to pay a levy to government for measures to [help smokers quit and prevent young people from taking up smoking/reduce and prevent obesity/reduce and prevent harm from alcohol/reduce and prevent harm from gambling]”. The bars in* Fig. 2*represent 95% confidence intervals.*

Support was higher for policies that targeted industry directly (levies) compared with those that increased product prices for consumers (taxes) (Fig. 2). For levies, support was again highest for those targeting the tobacco industry (strongly support 52.5%, 95% CI 51.6% to 53.4%; tend to support 24.6%, 95% CI 23.8% to 25.3%) and gambling industry (strongly support 50.7%, 95% CI 49.8% to 51.6%; tend to support 23.5%, 95% CI 22.7% to 24.3%). However, the majority of respondents also supported levies targeting unhealthy food and drinks manufacturers (strongly support 32.5%, 95% CI 31.6% to 33.3%; tend to support 27.0%, 95% CI 26.2% to 27.8%) and the alcohol drinks industry (strongly support 34.5%, 95% CI 33.7% to 35.4%; tend to support 26.6%, 95% CI 25.8% to 27.4%). In contrast, only taxes to raise the price of tobacco had majority support amongst respondents (strongly support 40.7%, 95% CI 39.8% to 41.6%; tend to support 20.7%, 95% CI 20.0% to 21.5%), although more respondents supported than opposed suggestions of tax to increase the prices of unhealthy food and drink (strongly support 19.3%, 95% CI 18.6% to 20.0%; tend to support 23.4%, 95% CI 22.6% to 24.2%; tend to oppose 17.8%, 95% CI 17.1% to 18.5%, strongly oppose 14.7%, 95% CI 14.0% to 15.3%) and alcohol (strongly support 19.3%, 95% CI 18.5% to 20.0%; tend to support 19.1%, 95% CI 18.3% to 19.8%; tend to oppose 18.8%, 95% CI 18.1% to 19.5%, strongly oppose 15.4%, 95% CI 14.8% to 16.1%). There was also majority support for extending the existing sugar tax to include other categories of high-sugar foods (strongly support 26.2%, 95% CI 25.4% to 27.0%; tend to support 26.8%, 95% CI 26.0% to 27.6%).

#### Marketing restrictions

**Fig. 3:**
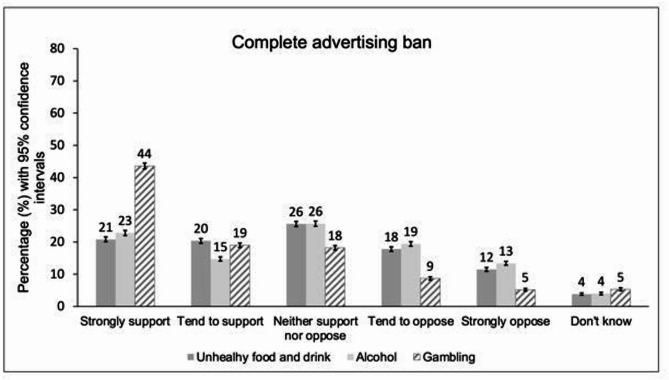
Percentage support around complete advertising bans across unhealthy commodity industries. Legend: The figure shows support for statements: *“complete ban on all [unhealthy food and drink/alcohol/gambling] advertising” (Weighted*,* N*_*unweighted*_*= 12 271). The bars in* Fig. 3*represent 95% confidence intervals.*

Respondents gave more mixed responses around marketing restrictions. The majority supported a complete gambling advertising ban (strongly support 43.6%, 95% CI 42.7% to 44.5%; tend to support 19.0%, 95% CI 18.3% to 19.7%). However, there was less certainty around complete bans on alcohol advertising (strongly support 22.8%, 95% CI 22.0% to 23.6%; tend to support 14.7%, 95% CI 14.1% to 15.4%) and unhealthy food advertising (strongly support 20.8%, 95% CI 20.1% to 21.6%; tend to support 20.4%, 95% CI 19.6% to 21.1%); although support did outweigh opposition in both cases (Fig. 3) and there was majority support for many individual marketing restrictions (see supplementary material). As there are already near-complete bans on tobacco marketing, participants were not asked around their support for a ban on tobacco advertising.

#### Health warnings

**Fig. 4:**
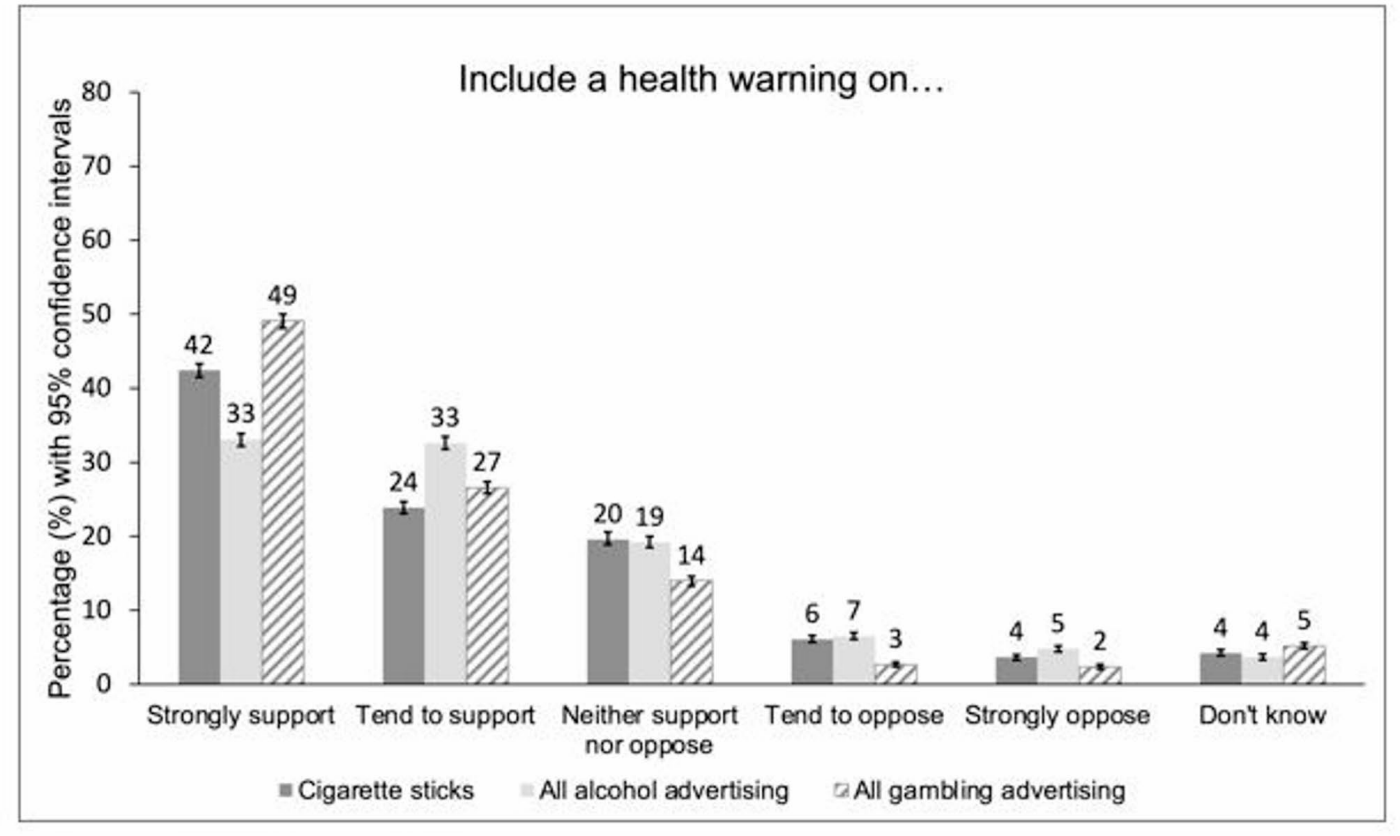
Percentage support around health warnings across unhealthy commodity industries. Legend: Percentage support for statements: *“heath warnings printed on cigarette sticks to encourage smokers to quit/including a health warning on all [alcohol/gambling] advertising to communicate the risks associated with [drinking alcohol/gambling]”*. Weighted, N_unweighted_= 12 271. *The bars in* Fig. 4*represent 95% confidence intervals.*

Health warnings were popular amongst respondents with 66.3% supporting them on cigarette sticks (strongly support 42.4%, 95% CI 41.5% to 43.3%; tend to support 23.9%, 95% CI 23.1% to 24.7%); 65.7% supporting them on all alcohol advertising (strongly support 33.1%, 95% CI 32.2% to 33.9%; tend to support 32.6%, 95% CI 31.8% to 33.5%) and 75.7% supporting them on all gambling advertising (strongly support 49.1%, 95% CI 48.1% to 50.0%; tend to support 26.6%, 95% CI 25.8% to 27.4%) (Fig. 4).

#### Age restrictions for tobacco and gambling

The vast majority of respondents (87.4%) supported having a minimum age of 18 for all forms of gambling (strongly support 67.4%, 95% CI 66.6% to 68.3%; tend to support 20.0%, 95% CI 19.3% to 20.7%). For tobacco, the majority of respondents (64.3%) supported ‘increasing the age of sale from 18 to 21’ (strongly support 43.1%, 95% CI 42.2% to 44.0%; tend to support 21.2%, 95% CI 20.4% to 21.9%), while ‘increasing the age of sale every year by one year, until no one can buy a tobacco product’ was supported by 49.5%, compared to 24.9% who opposed it (strongly support 32.3%, 95% CI 31.4% to 33.1%; tend to support 17.2%, 95% CI 16.6% to 17.9%; tend to oppose 13.2%, 95% CI 12.6% to 13.8%; strongly oppose 11.7%, 95% CI 11.1% to 12.3%).

### Factors associated with support for policies

Sociodemographic characteristics and consumption of unhealthy commodities were associated with differing levels of support for public health policies on tobacco control (see supplementary material for full results).

#### Sociodemographic characteristics

There were small differences between men and women in the odds of supporting public health policies across commodities. Where such differences were seen, women tended to be more supportive of public health policies than men. There were exceptions including protecting public health policy from the influence of industry and representatives for tobacco (OR_adj_ 0.93, 95% CI 0.92 to 0.95 and unhealthy food and drinks (OR_adj_ 0.97, 95% CI 0.95 to 0.99). Women had also slightly lower odds of supporting using tax to increase the price of unhealthy food and drink (OR_adj_ 0.97, 95% CI 0.95 to 0.99).

Generally, older age groups were more likely to support public health policies than the youngest age group (18- to 24- year-olds). For almost all policies, the oldest age group (aged 65 years and over) had the highest odds of supporting the policies compared with the youngest age group. The observed associations between age and level of support are less pronounced for tobacco and alcohol policies compared with gambling and unhealthy food and drink policies. This was particularly true for the following policies, which were equally supported across age groups: increasing age of sale of tobacco one year every year, requiring health warnings on cigarettes, and ensuring that alcohol display and promotion in shops and supermarkets is only visible to people intending to browse or purchase alcohol.

Concerning social grade, less advantaged social grades (C2DE) had lower odds of supporting most policies compared with more advantaged social grades (ABC1). The one exception was for a complete advertising ban for alcoholic drinks where there was no difference in support between social grades C2DE and ABC1 (OR_adj_ 1.00, 95% CI 0.98 to 1.02).

#### Harmful consumption of unhealthy commodities

Current smoking reduced the odds of supporting policies across all commodities, and this was particularly marked for tobacco control policies. The two policies with the biggest difference in support between smokers and non-smokers were related to tax increases and health warnings on cigarette sticks: smokers had 36% lower odds of supporting using tax to increase the price of tobacco products by 5% above the rate of inflation each year than non-smokers (OR_adj_ 0.64, 95% CI 0.62 to 0.65) and 27% lower odds of supporting health warnings on cigarette sticks (OR_adj_ 0.73, 95% CI 0.71 to 0.75).

There were no meaningful associations between obesity (BMI ≥ 30) and the level of support for public health policies across all commodities.

Drinking alcohol above guideline recommendations was associated with a reduced likelihood of support for alcohol and gambling policies, and a slight reduction in support for most tobacco control policies and for some unhealthy food and drink policies. The biggest difference in support was related to tax: people consuming alcohol above the recommended guidelines had 16% lower odds of supporting using tax to increase the price of alcohol (OR_adj_ 0.84, 95% CI 0.82 to 0.86) compared with those not exceeding alcohol guideline recommendations.

Daily gambling was associated with reduced odds of support for most gambling policies, including complete advertising bans (OR_adj_ 0.85, 95% CI 0.79 to 0.93) and health warnings on all gambling advertising (OR_adj_ 0.88, 95% CI 0.81 to 0.95). In contrast with harmful consumption of alcohol and smoking, daily gambling was associated with slightly increased odds of supporting some alcohol and unhealthy food and drink policies. These policies included using tax to increase the price of unhealthy food and drink (OR_adj_ 1.09, 95% CI 1.00 to 1.18) and alcohol (OR_adj_ 1.13, 95% CI 1.04 to 1.22), and complete advertising bans for unhealthy food and drink (OR_adj_ 1.11, 95% CI 1.02 to 1.21) and alcoholic drinks (OR_adj_ 1.18, 95% CI 1.09 to 1.28).

## Discussion

### Summary of key findings

Overall, respondents supported most public health policies to reduce harm from tobacco, alcohol, unhealthy food and drink and gambling. However, support was comparatively higher for policies targeting tobacco and gambling. Notably, support was strong for measures targeting industry and its interaction with government, with high levels of support for protecting health policy from industry influence, for organisations submitting evidence to government and parliamentary committees to declare their funding, and for levies on tobacco, gambling and alcohol industries and unhealthy food and drink manufacturers.

The study identified differences in support for public health policies based on sociodemographic characteristics and the harmful consumption of unhealthy commodities. Individuals from less advantaged social grades were generally less supportive of policies, while small differences were observed regarding sex and age. Additionally, those consuming unhealthy commodities, or living with the consequences of them, were less supportive of policies targeting those specific commodities. The exception was those living with obesity, who showed only modest reduced support for some unhealthy food and drink policies and no difference in the level of support for tobacco, gambling or alcohol policies.

### Strengths and limitations of the study

This study is unique in its investigation of policy areas relating to four unhealthy commodities, and in its examination of the impact of harmful consumption of these commodities on public support across these policy areas. Also new within this study were the questions exploring government interaction with the industries behind these commodities. The study benefitted from a large sample size (over 12,000 respondents), representative of the British adult population.

However, there were limitations to consider. Self-reported measures may not accurately determine harmful exposure to unhealthy products, potentially affecting the strength of associations found in this study. There was also missingness for some variables (see supplementary material), however, this was mitigated through multiple imputations. Additionally, data disaggregation by gender was not possible due to the weighting and sampling methods employed by YouGov to reflect the adult British population and match official statistics. However, from 2024, YouGov has started to include a more inclusive gender identity question in the Adult Smokefree survey [27].

Finally, although the surveys were conducted online, the weighting methodology was designed to adjust for individuals who do not use the internet. While the sampling frame does not constitute a random probability sample, it is structured to produce a balanced and broadly representative sample.

### Comparison with previous research and interpretation of findings

The findings of this study are largely consistent with other studies. A systematic review of 200 studies by Diepeveen et al. [36] also found generally high support for policies targeting smoking and higher than for those targeting alcohol, diet, or physical activity. Furthermore, they reported that women and older people expressed higher support for policy interventions than men and younger people. Similar findings were reported by Kock et al. [37] and Reynolds et al. [38]. In the present study, people with less advantaged social grades generally tended to show lower support for policies than those from more advantaged social grades. A cross-sectional study of adults living in Great Britain in 2016 showed similar trends in support for policies targeting obesity [39]. Other studies found more mixed results for differences by socioeconomic position, but a clear tendency for lower support for smoking-related interventions by less advantaged social grades [36, 38]. One reason for these differences could be due to different measures used to define socioeconomic position.

The studies by Diepeveen et al. and Reynolds et al. also found that those engaging with unhealthy commodities were less supportive of interventions to stop the targeted behaviour compared with those who did not engage [36, 38], but these studies did not include gambling-related policies. Similarly, cross-sectional studies in Great Britain [37], England [40], and a Gambling Health Alliance poll [41] showed that smokers, those with increased levels of alcohol consumption and current gamblers, were less likely to support interventions targeting these unhealthy commodities, respectively. For unhealthy food and drink, however, the evidence was more mixed. Diepeeven et al. [36] reported mixed evidence around studies relating to diet and physical activity, with four out of six of the included studies also reporting no significant association between public acceptability of policies, diet and physical activity. However, Reynolds et al. [38] found that those eating more high-calorie snacks were less accepting of policies targeting this behaviour. These differences could be due to the studies using different measures related to unhealthy food and drink.

### Implications of findings

Remarkably high levels of support were identified for requiring organisations submitting evidence to government to declare their funding (88%) and for protecting health policy from the influence of health-harming industries. These findings are significant in considering new approaches to tackle unhealthy commodity industries, in which context measures to tackle conflicts of interest are recognised as being essential in underpinning innovative and effective approaches to health governance [14]. Particularly important here is the strength of public support for measures that clearly target unhealthy commodity industries. There was strong support for levies across all four policy areas, reflecting that that the logic of the “polluter pays” principle strongly resonates with the public. This provides a compelling case for action, particularly around alcohol and obesity, where support for other measures is less strong, and where related industries are a potential source for generating much needed funds for public health actions. Overall, the study findings should encourage government to consider upstream approaches targeting unhealthy commodity industries as they demonstrate policymakers can do so with confidence in the public’s support. While those who are impacted by unhealthy commodities, younger people, men and people of socio-economic grades C2DE are somewhat less likely to support these policies, differences in support for policy interventions across commodities are minor and, therefore, this should not deter policymakers. However, there may be implications for how policies should be communicated to those who consume unhealthy commodities and the need to specifically engage these groups through the policy making process. The findings of the present study provide a baseline for public support across four public health policy areas.

Future research should continue to monitor public support over time to increase the confidence of policymakers, and to establish if the adoption of innovative public health measures improves public support. Additionally, there could be an examination of the differences in public perception or level of concern across the four unhealthy commodities, which may have influenced participant responses to the original survey in this study. Finally, researchers could look to generate hypotheses with respect to the differential levels of public support for policies shown in this study, associated with age, social grade, and consumption of unhealthy commodities.

## Conclusions

This study found high levels of support amongst the public for protecting health policy from unhealthy commodity industries and for levies on industry to fund public health activities. Majority support was found for most suggested policies, with particularly high support for gambling and tobacco control policies. While differences were identified by sex, age, socioeconomic grade and harmful exposure to tobacco, alcohol and gambling, these differences were generally small and should not discourage policymakers.

## Supplementary Information

Additional file 1. Missing data tables, including full questions for those included in odds ratios table and support for these.

Additional file 2. STROBE checklist.

## Data Availability

The datasets used and/or analysed during the current study are available upon reasonable request from the corresponding author.
