# Supplementary material for: Public opinion on policy interventions for regulating four unhealthy commodity industries: a cross-sectional online survey of a representative sample of British adults 2023
Source: BMC Public Health. 2025 Nov 21;25:4402. doi: 10.1186/s12889-025-25271-x (PMC12754964; doi:10.1186/s12889-025-25271-x)
Supplement: Supplementary file 1 — Additional file 1. Missing data tables, including full questions for those included in odds ratios table and support for these. [file 12889_2025_25271_MOESM1_ESM.docx]

**Missing data**

Table 1: Number and percent of missing values for each variable of interest

| **Variable** | **Missing values n (%)** |
| --- | --- |
| Age | 0 (0) |
| Gender | 0 (0) |
| Body mass index | 1912 (15.6) |
| Social grade | 0 (0) |
| Vote in past election | 34 (0.3) |
| Smoking status | 0 (0) |
| Alcohol consumption level | 898 (7.3) |
| Gambling status | 0 (0) |
| Smoking policy support statements | 0 (0) |
| Food policy support statements | 0 (0) |
| Alcohol policy support statements | 0 (0) |
| Gambling policy support statements | 0 (0) |

**General question**

Table 2: How strongly, if at all, do you agree or disagree with the following statement...? Organisations submitting evidence to government and parliamentary committees should be required to declare who they get their funding from (weighted)

| **Funding declaration required** | **% (95% CI)** |
| --- | --- |
| Strongly agree | 59.9 (59.0, 60.8) |
| Agree | 28.0 (27.2, 28.9) |
| Neither agree nor disagree | 6.1 (5.7, 6.6) |
| Disagree | 0.6 (0.5, 0.8) |
| Strongly disagree | 0.2 (0.1, 0.3) |
| Don’t know | 5.1 (4.7, 5.5) |

**Tobacco control policies**

Table 3: How strongly, if at all, do you support or oppose the following measures…? All Government health policy should be protected from the influence of the tobacco industry and its representatives

| **Protect policy tobacco** | **% (95% CI)** |
| --- | --- |
| Strongly support | 55.0 (54.1, 55.9) |
| Tend to support | 20.0 (19.3, 20.7) |
| Neither support nor oppose | 10.4 (9.9, 11.0) |
| Tend to oppose | 1.5 (1.3, 1.8) |
| Strongly oppose | 0.9 (0.8, 1.1) |
| Don’t know | 12.1 (11.5, 12.7) |

Table 4: How strongly, if at all, do you support or oppose the following measures…? Tax should be used to increase the price of tobacco products 5% above the rate of inflation each year

| **Tax above inflation** | **% (95% CI)** |
| --- | --- |
| Strongly support | 40.7 (39.8, 41.6) |
| Tend to support | 20.7 (20.0, 21.5) |
| Neither support nor oppose | 16.2 (15.5, 16.9) |
| Tend to oppose | 8.5 (8.0, 9.0) |
| Strongly oppose | 8.8 (8.3, 9.4) |
| Don’t know | 5.1 (4.6, 5.5) |

Table 5: How strongly, if at all, do you support or oppose the following measures…? Requiring tobacco manufacturers to pay a levy to Government for measures to help smokers quit and prevent young people from taking up smoking

| **Levy** | **% (95% CI)** |
| --- | --- |
| Strongly support | 52.5 (51.6, 53.4) |
| Tend to support | 24.6 (23.8, 25.3) |
| Neither support nor oppose | 12.1 (11.5, 12.7) |
| Tend to oppose | 3.5 (3.2, 3.8) |
| Strongly oppose | 2.7 (2.4, 3.0) |
| Don’t know | 4.6 (4.2, 5.0) |

Table 6: How strongly, if at all, do you support or oppose the following measures…? Raise the age of sale from 18 to 21 for tobacco

| **Age of sale** | **% (95% CI)** |
| --- | --- |
| Strongly support | 43.1 (42.2, 44.0) |
| Tend to support | 21.2 (20.4, 21.9) |
| Neither support nor oppose | 17.8 (17.1, 18.5) |
| Tend to oppose | 8.6 (8.1, 9.1) |
| Strongly oppose | 5.5 (5.1, 6.0) |
| Don’t know | 3.8 (3.5, 4.2) |

Table 7: How strongly, if at all, do you support or oppose the following measures…? Increase the age of sale from 18, by one year, every year until no one can buy a tobacco product in this country

| **Raise age of sale 1 year every year** | **% (95% CI)** |
| --- | --- |
| Strongly support | 32.3 (31.4, 33.1) |
| Tend to support | 17.2 (16.6, 17.9) |
| Neither support nor oppose | 19.5 (18.8, 20.2) |
| Tend to oppose | 13.2 (12.6, 13.8) |
| Strongly oppose | 11.7 (11.1, 12.3) |
| Don’t know | 6.0 (5.6, 6.5) |

Table 8: How strongly, if at all, do you support or oppose the following measures…? Require cigarette packs to include inserts with Government information about quitting

| **Pack inserts** | **% (95% CI)** |
| --- | --- |
| Strongly support | 38.7 (37.8, 39.6) |
| Tend to support | 28.2 (27.4, 29.0) |
| Neither support nor oppose | 19.9 (19.1, 20.6) |
| Tend to oppose | 5.5 (5.1, 6.0) |
| Strongly oppose | 3.7 (3.3, 4.0) |
| Don’t know | 4.0 (3.6, 4.4) |

Table 9: How strongly, if at all, do you support or oppose the following measures…? Increased Government investment in public education campaigns on smoking aimed at adults and children

| **Public education campaigns** | **% (95% CI)** |
| --- | --- |
| Strongly support | 38.5 (37.6, 39.4) |
| Tend to support | 31.8 (31.0, 32.7) |
| Neither support nor oppose | 18.1 (17.4, 18.8) |
| Tend to oppose | 4.3 (4.0, 4.7) |
| Strongly oppose | 2.9 (2.6, 3.2) |
| Don’t know | 4.3 (.0, 4.7) |

Table 10: How strongly, if at all, do you support or oppose the following measures…? Health warnings printed on cigarette sticks to encourage smokers to quit

| **Health warnings on cigarettes** | **% (95% CI)** |
| --- | --- |
| Strongly support | 42.4 (41.5, 43.3) |
| Tend to support | 23.9 (23.1, 24.7) |
| Neither support nor oppose | 19.6 (18.9, 20.6) |
| Tend to oppose | 6.1 (5.7, 6.6) |
| Strongly oppose | 3.7 (3.3, 4.0) |
| Don’t know | 4.2 (3.9, 4.7) |

Table 11: How strongly, if at all, do you support or oppose the following measures…? Smoking should be banned in all cars

| **Banned in cars** | **% (95% CI)** |
| --- | --- |
| Strongly support | 48.0 (47.1, 48.9) |
| Tend to support | 17.6 (16.9, 18,3) |
| Neither support nor oppose | 15.1 (14.4, 15.7) |
| Tend to oppose | 8.5 (8.0, 9.0) |
| Strongly oppose | 7.6 (7.1, 8.1) |
| Don’t know | 3.3 (2.9, 3.6) |

Table 12: How strongly, if at all, do you support or oppose the following measures…? Requiring businesses to have a valid licence to sell tobacco which can be removed if they are caught more than once selling to underage smokers

| **Licencing** | **% (95% CI)** |
| --- | --- |
| Strongly support | 58.3 (57.4, 59.3) |
| Tend to support | 24.9 (24.1, 25.7) |
| Neither support nor oppose | 9.1 (8.5, 9.6) |
| Tend to oppose | 2.3 (2.1, 2.6) |
| Strongly oppose | 1.8 (1.5, 2.0) |
| Don’t know | 3.5 (3.2, 3.9) |

Table 13: Overall support and adjusted odds ratios^1^ for support of tobacco control policies (weighted, N_unweighted_=12,271).

| **Policy** | **Overall support (%)** | **Age (ref: 18-24)** | | | | | **Sex (ref: men)** | **Social grade (ref: ABC1)** | **Currently smoking (ref: no)** | **BMI ≥30 (ref: no)** | **Drinking alcohol above guidelines (ref: no)** | **Gambling daily (ref: no)** |
| --- | --- | --- | --- | --- | --- | --- | --- | --- | --- | --- | --- | --- |
|  |  | 25-34 | 35-44 | 45-54 | 55-64 | 65+ | Women | C2DE | Yes | Yes | Yes | Yes |
| Health policy should be protected from the influence of the tobacco industry | 75.0 | 1.06 (1.03, 1.10) | 1.09 (1.06, 1.13) | 1.12 (1.08, 1.16) | 1.19 (1.16, 1.23) | 1.21 (1.17, 1.25) | 0.93 (0.92, 0.95) | 0.90 (0.89, 0.92) | 0.82 (0.80, 0.84) | 0.99 (0.97, 1.00) | 1.00 (0.98, 1.02) | 1.00 (0.93, 1.08) |
| Tax to raise price 5% above inflation | 61.4 | 0.98 (0.94, 1.01) | 1.00 (0.97, 1.04) | 1.01 (0.97, 1.04) | 1.05 (1.02, 1.09) | 1.08 (1.05, 1.12) | 1.02 (1.00, 1.04) | 0.91 (0.90, 0.93) | 0.64 (0.62, 0.65) | 1.01 (0.99, 1.03) | 0.95 (0.93, 0.98) | 1.05 (0.96, 1.13) |
| Levy on tobacco manufacturers to pay for measures to help smokers quit and prevent young people taking up smoking | 77.1 | 1.04 (1.00, 1.07) | 1.06 (1.03, 1.10) | 1.09 (1.06, 1.13) | 1.12 (1.08, 1.15) | 1.12 (1.08, 1.15) | 1.02 (1.00, 1.04) | 0.92 (0.90, 0.93) | 0.78 (0.76, 0.80) | 1.00 (0.98. 1.01) | 0.97 (0.95, 0.99) | 0.97 (0.91. 1.05) |
| Raise the age of sale from 18 to 21 | 64.3 | 1.04 (1.00, 1.07) | 1.07 (1.04, 1.11) | 1.06 (1.02, 1.10) | 1.05 (1.01, 1.09) | 1.07 (1.03, 1.10) | 1.04 (1.02, 1.06) | 0.96 (0.94, 0.98) | 0.83 (0.81, 0.85) | 0.99 (0.97, 1.01) | 0.93 (0.91, 0.95) | 1.03 (0.95. 1.11) |
| Age of sale to increase 1 year every year | 49.5 | 0.98 (0.94, 1.01) | 1.01 (0.97, 1.04) | 1.00 (0.96, 1.04) | 0.99 (0.95, 1.02) | 1.00 (0.97, 1.04) | 0.99 (0.98, 1.01) | 0.96 (0.95, 0.98) | 0.81 (0.78, 0.83) | 1.00 (0.98, 1.02) | 0.95 (0.93, 0.98) | 1.02 (0.93, 1.11) |
| Pack inserts with information about quitting | 66.9 | 0.97 (0.93, 1.00) | 0.99 (0.95, 1.02) | 1.02 (0.98, 1.05) | 1.06 (1.02, 1.09) | 1.07 (1.03, 1.1) | 1.01 (1.00, 1.03) | 0.94 (0.92, 0.96) | 0.77 (0.74, 0.79) | 1.00 (0.98, 1.02) | 0.94 (0.92, 0.96) | 0.99 (0.91, 1.07) |
| Increased investment in education campaigns | 70.3 | 0.97 (0.94, 1.01) | 0.98 (0.95, 1.02) | 0.99 (0.96, 1.03) | 1.03 (0.99, 1.06) | 1.04 (1.01, 1.07) | 1.04 (1.02, 1.05) | 0.93 (0.91, 0.94) | 0.83 (0.81, 0.86) | 0.99 (0.97, 1.01) | 0.97 (0.95, 1.00) | 1.01 (0.93, 1.09) |
| Health warnings on cigarette sticks to encourage smokers to quit | 66.3 | 0.97 (0.94, 1.01) | 0.97 (0.94, 1.00) | 0.99 (0.95, 1.02) | 0.99 (0.95, 1.02) | 1.01 (0.98, 1.04) | 1.00 (0.98, 1.02) | 0.95 (0.93, 0.96) | 0.73 (0.71, 0.75) | 0.98 (0.96, 1.00) | 0.94 (0.92, 0.96) | 1.02 (0.94, 1.10) |
| Ban smoking in all in cars | 65.5 | 0.96 (0.92, 0.99) | 0.98 (0.95, 1.01) | 0.98 (0.95, 1.02) | 1.02 (0.99, 1.06) | 1.08 (1.05, 1.11) | 1.07 (1.05, 1.08) | 0.95 (0.93, 0.96) | 0.70 (0.68, 0.72) | 1.01 (0.99, 1.03) | 0.92 (0.91, 0.95) | 1.03 (0.95, 1.11) |
| Requiring businesses to have a valid licence to sell tobacco which can be removed if they are caught selling more than once to underage smokers | 83.3 | 1.02 (0.99, 1.06) | 1.04 (1.01, 1.07) | 1.07 (1.04, 1.11) | 1.11 (1.08, 1.14) | 1.12 (1.09, 1.15) | 1.03 (1.01, 1.04) | 0.94 (0.92, 0.95) | 0.86 (0.84, 0.76) | 1.01 (0.99, 1.02) | 0.97 (0.95, 0.99) | 0.93 (0.87, 1.00) |

^1^Respective other variables were used for adjustment.

**Unhealthy food and drink policies**

Table 14: How strongly, if at all, would you support or oppose the following measure…? All government health policy should be protected from the influence of unhealthy food and drink manufacturers, and their representatives

| **Policy protected** | **% (95% CI)** |
| --- | --- |
| Strongly support | 45.1 (44.2, 46.0) |
| Tend to support | 23.1 (22.3, 23.8) |
| Neither support nor oppose | 17.8 (17.1, 18.5) |
| Tend to oppose | 4.4 (4.0, 4.7) |
| Strongly oppose | 3.2 (2.8, 3.5) |
| Don’t know | 6.5 (6.0, 7.0) |

Table 15: How strongly, if at all, do you support or oppose the following? Tax should be used to increase the price of unhealthy food and drink

| **Tax to increase price of unhealthy food** | **% (95% CI)** |
| --- | --- |
| Strongly support | 19.3 (18.6, 20.0) |
| Tend to support | 23.4 (22.6, 24.2) |
| Neither support nor oppose | 20.8 (20.1, 21.5) |
| Tend to oppose | 17.8 (17.1, 18.5) |
| Strongly oppose | 14.7 (14.0, 15.3) |
| Don’t know | 4.0 (3.6, 4.4) |

Table 16: The Soft Drink Industry Levy (SDIL), also known as the 'Sugar Tax', means that the manufacturers of soft drinks have to pay more tax depending on the amount of sugar that goes into their products. The tax applies to the manufacturers but they may increase the cost of drinks in shops as a result. How strongly, if at all, would you support or oppose the following measure…? Extend the existing sugar tax to include other categories of high-sugar foods

| **Sugar tax** | **% (95% CI)** |
| --- | --- |
| Strongly support | 26.2 (25.4, 27.0) |
| Tend to support | 26.8 (26.0, 27.6) |
| Neither support nor oppose | 18.3 (17.5, 19.0) |
| Tend to oppose | 12.1 (11.5, 12.7) |
| Strongly oppose | 11.8 (11.2, 12.4) |
| Don’t know | 4.8 (4.4, 5.2) |

Table 17: How strongly, if at all, would you support or oppose the following measure…? Requiring unhealthy food and drink manufacturers to pay a levy to government for measures to reduce and prevent obesity

| **Levy** | **% (95% CI)** |
| --- | --- |
| Strongly support | 32.5 (31.6, 33.3) |
| Tend to support | 27.0 (26.2, 27.8) |
| Neither support nor oppose | 20.1 (19.3, 20.8) |
| Tend to oppose | 8.6 (8.1, 9.1) |
| Strongly oppose | 6.7 (6.3, 7.2) |
| Don’t know | 5.2 (4.7, 5.6) |

Table 18: How strongly, if at all, would you support or oppose the following measure…? Complete ban on all unhealthy food advertising

| **Unhealthy food advertising ban** | **% (95% CI)** |
| --- | --- |
| Strongly support | 20.8 (20.1, 21.6) |
| Tend to support | 20.4 (19.6, 21.1) |
| Neither support nor oppose | 25.6 (24.8, 26.4) |
| Tend to oppose | 17.8 (17.1, 18.5) |
| Strongly oppose | 11.5 (10.9, 12.1) |
| Don’t know | 3.8 (3.5, 4.2) |

Table 19: How strongly, if at all, would you support or oppose the following measure…? Only adverts for healthier food and drinks being shown on social media and online

| **Online advertising** | **% (95% CI)** |
| --- | --- |
| Strongly support | 24.1 (23.3,24.9) |
| Tend to support | 25.9 (25.1, 26.7) |
| Neither support nor oppose | 25.4 (24.6, 26.2) |
| Tend to oppose | 12.7 (12.1, 13.3) |
| Strongly oppose | 7.7 (7.2, 8.2) |
| Don’t know | 4.2 (3.8, 4.6) |

Table 20: How strongly, if at all, would you support or oppose the following measure…? Stopping brands that sell unhealthy food from sponsoring sports events and teams

| **Restrictions on in-store displays** | **% (95% CI)** |
| --- | --- |
| Strongly support | 27.8 (27.0, 28.6) |
| Tend to support | 24.6 (23.8, 25.4) |
| Neither support nor oppose | 23.6 (22.9, 24.4) |
| Tend to oppose | 12.2 (11.6, 12.8) |
| Strongly oppose | 7.7 (7.2, 8.2) |
| Don’t know | 4.1 (3.7, 4.4) |

Table 21: How strongly, if at all, would you support or oppose the following measure…? Restrictions on placing unhealthy foods in prominent areas such as checkouts and aisle ends

| **Restrictions on in-store displays** | **% (95% CI)** |
| --- | --- |
| Strongly support | 33.4 (32.6, 34.3) |
| Tend to support | 30.6 (29.7, 31.4) |
| Neither support nor oppose | 18.4 (17.6, 19.1) |
| Tend to oppose | 8.0 (7.5, 8.5) |
| Strongly oppose | 6.0 (5.5, 6.4) |
| Don’t know | 3.6 (3.3, 4.0) |

Table 22: How strongly, if at all, would you support or oppose the following measure…? Only adverts for healthier food and drinks being shown on TV and radio before 9pm

| **Watershed on unhealthy food advertising** | **% (95% CI)** |
| --- | --- |
| Strongly support | 27.8 (27.0, 28.6) |
| Tend to support | 28.6 (27.7, 29.4) |
| Neither support nor oppose | 22.4 (21.6, 23.2) |
| Tend to oppose | 10.4 (9.8, 10.9) |
| Strongly oppose | 6.8 (6.3, 7.3) |
| Don’t know | 4.1 (3.7, 4.5) |

Table 23: How strongly, if at all, would you support or oppose the following measure…? Government to require food manufacturers to reduce sugar in food

| **Reduce sugar** | **% (95% CI)** |
| --- | --- |
| Strongly support | 31.1 (30.3, 32.0) |
| Tend to support | 32.3 (31.4, 33.1) |
| Neither support nor oppose | 17.3 (16.6, 18.0) |
| Tend to oppose | 9.0 (8.5, 9.5) |
| Strongly oppose | 6.9 (6.5, 7.4) |
| Don’t know | 3.4 (3.1, 3.8) |

Table 24: How strongly, if at all, would you support or oppose the following measure…? Government to require food manufacturers to reduce calories in food

| **Reduce calories** | **% (95% CI)** |
| --- | --- |
| Strongly support | 23.1 (22.3, 23.8) |
| Tend to support | 27.4 (26.5, 28.2) |
| Neither support nor oppose | 24.3 (23.6, 25.1) |
| Tend to oppose | 12.2 (11.6, 12.8) |
| Strongly oppose | 8.9 (8.4, 9.5) |
| Don’t know | 4.1 (3.7, 4.5) |

Table 25: Overall support and adjusted odds ratios^1^ for support of unhealthy food and drink policies (weighted, N_unweighted_=12,271)

| **Policy** | **Overall support (%)** | **Age (ref: 18-24)** | | | | | **Sex (ref: men)** | **Social grade (ref: ABC1)** | **Currently smoking (ref: no)** | **BMI ≥30 (ref: no)** | **Drinking alcohol above guidelines (ref: no)** | **Gambling daily (ref: no)** |
| --- | --- | --- | --- | --- | --- | --- | --- | --- | --- | --- | --- | --- |
|  |  | 25-34 | 35-44 | 45-54 | 55-64 | 65+ | Women | C2DE | Yes | Yes | Yes | Yes |
| Health policy should be protected from the influence of unhealthy food and drink manufacturers | 68.2 | 1.04 (1.00, 1.07) | 1.06 (1.03, 1.10) | 1.08 (1.05, 1.12) | 1.14 (1.10, 1.18) | 1.17 (1.13, 1.21) | 0.97 (0.95, 0.99) | 0.91 (0.89, 0.92) | 0.89 (0.87, 0.92) | 0.99 (0.97, 1.01) | 0.99 (0.97, 1.01) | 1.01 (0.93, 1.09) |
| Tax to increase price of unhealthy food and drink | 42.7 | 0.98 (0.95, 1.02) | 1.00 (0.97, 1.04) | 1.00 (0.97, 1.04) | 1.06 (1.02, 1.10) | 1.11 (1.07, 1.15) | 0.97 (0.95, 0.99) | 0.90 (0.89, 0.92) | 0.92 (0.88, 0.95) | 0.97 (0.95, 0.99) | 1.00 (0.97, 1.02) | 1.09 (1.00, 1.18) |
| Extend existing sugar tax to include other high-sugar foods | 53.0 | 0.98 (0.95, 1.02) | 1.03 (0.99, 1.06) | 1.02 (0.99, 1.06) | 1.09 (1.06, 1.14) | 1.19 (1.15, 1.23) | 1.00 (0.99, 1.02) | 0.91 (0.90, 0.93) | 0.89 (0.86, 0.91) | 1.00 (0.98, 1.03) | 1.01 (0.99, 1.04) | 1.08 (0.99, 1.17) |
| Levy on unhealthy food and drink manufacturers to reduce and prevent obesity | 59.5 | 1.03 (0.99, 1.06) | 1.04 (1.00, 1.08) | 1.04 (1.01, 1.08) | 1.10 (1.06, 1.14) | 1.14 (1.11, 1.18) | 1.01 (0.99, 1.03) | 0.90 (0.89, 0.92) | 0.89 (0.87, 0.92) | 0.99 (0.97, 1.01) | 0.98 (0.96, 1.00) | 1.06 (0.98, 1.15) |
| Complete advertising ban on all unhealthy food | 41.2 | 1.04 (1.01, 1.08) | 1.06 (1.02, 1.09) | 1.07 (1.03, 1.11) | 1.13 (1.09, 1.17) | 1.22 (1.18, 1.26) | 1.02 (1.00, 1.03) | 0.96 (0.94, 0.98) | 0.95 (0.93, 0.98) | 0.99 (0.97, 1.01) | 0.97 (0.95, 0.99) | 1.11 (1.02, 1.21) |
| Only adverts for healthier food and drinks on social media and online | 50.0 | 1.07 (1.03, 1.11) | 1.08 (1.04, 1.12) | 1.07 (1.04, 1.11) | 1.12 (1.08, 1.16) | 1.20 (1.16, 1.24) | 1.05 (1.03, 1.07) | 0.95 (0.94, 0.97) | 0.92 (0.90, 0.95) | 0.99 (0.97, 1.01) | 0.96 (0.94, 0.98) | 1.10 (1.01, 1.19) |
| Ban on brands selling unhealthy food from sponsoring sports events and teams | 52.4 | 1.06 (1.02, 1.10) | 1.09 (1.05, 1.13) | 1.11 (1.07, 1.15) | 1.14 (1.10, 1.19) | 1.23 (1.19, 1.23) | 1.03 (1.01, 1.05) | 0.92 (0.91, 0.94) | 0.92 (0.89, 0.94) | 0.98 (0.96, 1.00) | 0.98 (0.96, 1.01) | 0.98 (0.90, 1.06) |
| Restrictions on placing unhealthy food in prominent areas such as checkouts and aisle ends | 64.0 | 1.05 (1.01, 1.09) | 1.08 (1.04, 1.12) | 1.11 (1.07, 1.15) | 1.16 (1.12, 1.20) | 1.22 (1.18, 1.26) | 1.08 (1.06, 1.10) | 0.91 (0.90, 0.93) | 0.90 (0.87, 0.92) | 1.00 (0.98, 1.02) | 0.98 (0.96, 1.00) | 1.03 (0.95, 1.12) |
| Only adverts for healthier food and drinks on TV and radio before 9pm | 56.3 | 1.06 (1.02, 1.10) | 1.10 (1.06, 1.14) | 1.12 (1.08, 1.16) | 1.17 (1.13, 1.21) | 1.25 (1.21, 1.29) | 1.05 (1.03, 1.06) | 0.93 (0.91, 0.95) | 0.93 (0.90, 0.95) | 0.98 (0.96, 1.00) | 0.96 (0.94, 0.98) | 1.01 (0.93, 1.10) |
| Require manufacturers to reduce sugar in food | 63.4 | 1.05 (1.01, 1.09) | 1.06 (1.03, 1.10) | 1.11 (1.07, 1.15) | 1.17 (1.13, 1.22) | 1.24 (1.20, 1.29) | 1.03 (1.02, 1.05) | 0.92 (0.91, 0.94) | 0.91 (0.89, 0.94) | 1.01 (0.99, 1.03) | 0.96 (0.94, 0.98) | 1.01 (0.93, 1.09) |
| Require manufacturers to reduce calories in food | 50.4 | 1.00 (0.96, 1.04) | 1.01 (0.97, 1.04) | 1.06 (1.02, 1.10) | 1.07 (1.03, 1.11) | 1.11 (1.07, 1.15) | 1.04 (1.02, 1.06) | 0.96 (0.94, 0.98) | 0.93 (0.91, 0.96) | 1.03 (1.01, 1.05) | 0.96 (0.94, 0.99) | 1.07 (0.98, 1.16) |

^1^Respective other variables were used for adjustment.

**Alcohol policies**

Table 26: How strongly, if at all, would you support or oppose the following measure…? All government health policy should be protected from the influence of the alcohol industry and its representatives

| **Protect policy** | **% (95% CI)** |
| --- | --- |
| Strongly support | 47.9 (47.0, 48.8) |
| Tend to support | 21.9 (21.1, 22.7) |
| Neither support nor oppose | 17.3 (16.6, 18.0) |
| Tend to oppose | 3.4 (3.1, 3.8) |
| Strongly oppose | 2.9 (2.6, 3.3) |
| Don’t know | 6.5 (6.1, 7.0) |

Table 27: How strongly, if at all, would you support or oppose the following measure…?

- Requiring the alcoholic drinks industry to pay a levy to government for measures to reduce and prevent harm from alcohol
- Requiring the alcoholic drinks manufacturers to pay a levy to government for measures to reduce and prevent harm from alcohol*

| **Levy** | **% (95% CI)** |
| --- | --- |
| Strongly support | 34.5 (33.7, 35.4) |
| Tend to support | 26.6 (25.8, 27.4) |
| Neither support nor oppose | 19.6 (18.9, 20.4) |
| Tend to oppose | 7.7 (7.2, 8.2) |
| Strongly oppose | 6.1 (5.7, 6.5) |
| Don’t know | 5.5 (5.1, 5.9) |

*participants randomised to wording and combined for analysis.

Table 28: How strongly, if at all, do you support or oppose the following: Tax should be used to increase the price of alcohol

| **Alcohol tax** | **% (95% CI)** |
| --- | --- |
| Strongly support | 19.3 (18.5, 20.0) |
| Tend to support | 19.1 (18.3, 19.8) |
| Neither support nor oppose | 23.5 (22.7, 24.3) |
| Tend to oppose | 18.8 (18.1, 19.5) |
| Strongly oppose | 15.4 (14.8, 16.1) |
| Don’t know | 3.9 (3.6, 4.3) |

Table 29: How strongly, if at all, would you support or oppose the following measure…? Complete ban on all alcohol advertising

| **Advertising bans** | **% (95% CI)** |
| --- | --- |
| Strongly support | 22.8 (22.0, 23.6) |
| Tend to support | 14.7 (14.1, 15.4) |
| Neither support nor oppose | 25.7 (24.9, 26.5) |
| Tend to oppose | 19.4 (18.7, 20.1) |
| Strongly oppose | 13.4 (12.8, 14.0) |
| Don’t know | 4.0 (3.6, 4.4) |

Table 30: How strongly, if at all, would you support or oppose the following measure…? Not allowing alcohol advertising on TV and radio

| **TV and radio advertising ban** | **% (95% CI)** |
| --- | --- |
| Strongly support | 28.5 (27.7, 29.3) |
| Tend to support | 21.1 (20.4, 21.9) |
| Neither support nor oppose | 25.2 (24.4, 26.0) |
| Tend to oppose | 13.7 (13.0, 14.3) |
| Strongly oppose | 7.4 (7.0, 7.9) |
| Don’t know | 4.1 (3.7. 4.5) |

Table 31: How strongly, if at all, would you support or oppose the following measure…? Not allowing alcohol advertising in cinemas

| **Cinema advertising ban** | **% (95% CI)** |
| --- | --- |
| Strongly support | 30.2 (29.4, 31.0) |
| Tend to support | 23.0 (22.2, 23.7) |
| Neither support nor oppose | 24.3 (23.5, 25.1) |
| Tend to oppose | 11.7 (11.1, 12.3) |
| Strongly oppose | 6.6 (6.1, 7.0) |
| Don’t know | 4.2 (3.8, 4.6) |

Table 32: How strongly, if at all, would you support or oppose the following measure…? Not allowing alcohol companies to sponsor sporting events or teams

| **Sports sponsorship** | **% (95% CI)** |
| --- | --- |
| Strongly support | 30.4 (29.6, 31.3) |
| Tend to support | 20.9 (20.1, 21.6) |
| Neither support nor oppose | 23.1 (22.3, 23.8) |
| Tend to oppose | 13.2 (12.5, 13.8) |
| Strongly oppose | 8.3 (7.8, 8.8) |
| Don’t know | 4.2 (3.8, 4.6) |

Table 33: How strongly, if at all, would you support or oppose the following measure…? Not allowing alcohol advertising in outdoor and public spaces such as streets, parks and public transport

| **Outdoor advertising** | **% (95% CI)** |
| --- | --- |
| Strongly support | 30.3 (29.5, 31.1) |
| Tend to support | 24.1 (23.3, 24.9) |
| Neither support nor oppose | 23.8 (23.1, 24.6) |
| Tend to oppose | 11.0 (10.4, 11.6) |
| Strongly oppose | 6.5 (6.1, 7.0) |
| Don’t know | 4.2 (3.9, 4.6) |

Table 34: How strongly, if at all, would you support or oppose the following measure…? Not allowing alcohol advertising on social media and online

| **Online advertising** | **% (95% CI)** |
| --- | --- |
| Strongly support | 30.1 (29.3, 30.9) |
| Tend to support | 22.6 (21.8, 23.3) |
| Neither support nor oppose | 24.2 (23.4, 25.0) |
| Tend to oppose | 12.1 (11.5, 12.7) |
| Strongly oppose | 6.8 (6.3, 7.2) |
| Don’t know | 4.3 (3.9, 4.7) |

Table 35: How strongly, if at all, would you support or oppose the following measure…? Ensuring that alcohol display and promotion in shops and supermarkets is only visible to people intending to browse or purchase alcohol

| **In shop displays** | **% (95% CI)** |
| --- | --- |
| Strongly support | 30.3 (29.4, 31.1) |
| Tend to support | 30.2 (29.3, 31.0) |
| Neither support nor oppose | 21.3 (20.5, 22.0) |
| Tend to oppose | 8.1 (7.6, 8.6) |
| Strongly oppose | 5.8 (5.4, 6.2) |
| Don’t know | 4.4 (4.0, 4.8) |

Table 36: How strongly, if at all, would you support or oppose the following measure…? Including a health warning on all alcohol advertising to communicate the risks associated with drinking alcohol

| **Health warnings** | **% (95% CI)** |
| --- | --- |
| Strongly support | 33.1 (32.2, 33.9) |
| Tend to support | 32.6 (31.8, 33.5) |
| Neither support nor oppose | 19.2 (18.5, 20.0) |
| Tend to oppose | 6.5 (6.1, 7.0) |
| Strongly oppose | 4.8 (4.4, 5.2) |
| Don’t know | 3.7 (3.3, 4.1) |

Table 37: Overall support and adjusted odds ratios^1^ for support of alcohol policies (weighted, N_unweighted_=12,271)

| **Policy** | **Overall support (%)** | **Age (ref: 18-24)** | | | | | **Sex (ref: men)** | **Social grade (ref: ABC1)** | **Currently smoking (ref: no)** | **BMI ≥30 (ref: no)** | **Drinking alcohol above guidelines (ref: no)** | **Gambling daily (ref: no)** |
| --- | --- | --- | --- | --- | --- | --- | --- | --- | --- | --- | --- | --- |
|  |  | 25-34 | 35-44 | 45-54 | 55-64 | 65+ | Women | C2DE | Yes | Yes | Yes | Yes |
| Health policy should be protected from the influence of the alcohol industry | 69.8 | 1.05 (1.01, 1.09) | 1.06 (1.02, 1.09) | 1.09 (1.05, 1.13) | 1.13 (1.09, 1.17) | 1.16 (1.12, 1.20) | 0.98 (0.96, 1.00) | 0.91 (0.90, 0.93) | 0.89 (0.86, 0.91) | 0.99 (0.97, 1.01) | 0.94 (0.92, 0.96) | 1.02 (0.94, 1.10) |
| Levy on alcoholic drinks industry/manufacturers to pay for measures to reduce and prevent harm from alcohol | 61.6 | 1.01 (0.97, 1.04) | 1.03 (0.99, 1.06) | 1.03 (0.99, 1.07) | 1.04 (1.00, 1.08) | 1.08 (1.04, 1.11) | 1.05 (1.03, 1.07) | 0.91 (0.90, 0.93) | 0.89 (0.86, 0.91) | 1.00 (0.98, 1.02) | 0.90 (0.88, 0.92) | 1.07 (0.99, 1.16) |
| Tax to increase the price of alcohol | 38.3 | 0.97 (0.94, 1.01) | 0.98 (0.95, 1.02) | 0.96 (0.92, 0.99) | 0.96 (0.93, 1.00) | 0.98 (0.95, 1.02) | 1.04 (1.02, 1.06) | 0.96 (0.94, 0.98) | 0.90 (0.88, 0.93) | 1.02 (1.00, 1.04) | 0.84 (0.82, 0.86) | 1.13 (1.04, 1.22) |
| Complete ban on all alcohol advertising | 37.5 | 1.01 (0.98, 1.05) | 1.01 (0.98, 1.05) | 0.99 (0.96, 1.03) | 1.02 (0.99, 1.06) | 1.05 (1.02, 1.09) | 1.04 (1.02, 1.06) | 1.00 (0.98, 1.02) | 0.93 (0.91, 0.95) | 1.01 (0.99, 1.03) | 0.89 (0.87, 0.91) | 1.18 (1.09, 1.28) |
| Not allowing alcohol advertising on TV and radio | 49.6 | 1.01 (0.97, 1.05) | 1.00 (0.97, 1.04) | 1.02 (0.98, 1.05) | 1.06 (1.02, 1.10) | 1.12 (1.08, 1.15) | 1.07 (1.05, 1.09) | 0.97 (0.95, 0.99) | 0.91 (0.88, 0.93) | 1.00 (0.98, 1.02) | 0.89 (0.87, 0.91) | 1.08 (1.00, 1.18) |
| Not allowing alcohol advertising in cinemas | 53.2 | 0.98 (0.95, 1.02) | 0.99 (0.95, 1.02) | 0.99 (0.96, 1.03) | 1.00 (0.96, 1.04) | 1.05 (1.02, 1.09) | 1.08 (1.06, 1.10) | 0.97 (0.95, 0.99) | 0.89 (0.86, 0.91) | 1.00 (0.98, 1.02) | 0.87 (0.85, 0.89) | 1.02 (0.94, 1.10) |
| Not allowing alcohol companies to sponsor sporting events or teams | 51.3 | 1.00 (0.97, 1.04) | 1.02 (0.98, 1.06) | 1.04 (1.00, 1.08) | 1.06 (1.02, 1.10) | 1.09 (1.05, 1.12) | 1.07 (1.05, 1.09) | 0.95 (0.93, 0.97) | 0.91 (0.89, 0.94) | 1.00 (0.98, 1.02) | 0.90 (0.88, 0.92) | 1.01 (0.93, 1.10) |
| Not allowing alcohol advertising in outdoor and public spaces such as streets, parks and public transport | 54.4 | 0.99 (0.96, 1.03) | 1.01 (0.98, 1.05) | 1.01 (0.98, 1.05) | 1.04 (1.00, 1.08) | 1.10 (1.06, 1.14) | 1.08 (1.06, 1.10) | 0.96 (0.94, 0.98) | 0.88 (0.86, 0.90) | 1.00 (0.98, 1.02) | 0.88 (0.86, 0.90) | 1.05 (0.97, 1.15) |
| Not allowing alcohol advertising on social media and online | 52.7 | 1.02 (0.99, 1.06) | 1.04 (1.01, 1.08) | 1.05 (1.01, 1.09) | 1.10 (1.06, 1.14) | 1.18 (1.14, 1.22) | 1.06 (1.05, 1.08) | 0.96 (0.94, 0.98) | 0.89 (0.86, 0.91) | 1.01 (0.99, 1.03) | 0.88 (0.86, 0.90) | 1.02 (0.94, 1.10) |
| Ensuring that alcohol display and promotion in shops and supermarkets is only visible to people intending to browse or purchase alcohol | 60.4 | 0.97 (0.94, 1.01) | 0.97 (0.94, 1.01) | 0.97 (0.94, 1.01) | 0.97 (0.94, 1.00) | 1.00 (0.97, 1.03) | 1.08 (1.06, 1.10) | 0.94 (0.93, 0.96) | 0.90 (0.87, 0.92) | 1.01 (0.99, 1.03) | 0.89 (0.86, 0.91) | 1.04 (0.96, 1.12) |
| Include health warnings on all alcohol advertising to communicate the risks associated with drinking alcohol | 65.7 | 1.01 (0.97, 1.05) | 1.01 (0.98, 1.05) | 1.04 (1.00, 1.08) | 1.05 (1.02, 1.09) | 1.06 (1.03, 1.10) | 1.08 (1.06, 1.09) | 0.94 (0.92, 0.95) | 0.90 (0.87, 0.92) | 0.99 (0.97, 1.01) | 0.88 (0.86, 0.91) | 1.01 (0.93, 1.09) |

^1^Respective other variables were used for adjustment

**Gambling policies**

Table 38: How strongly, if at all, would you support the following measure…? All government health policy should be protected from the influence of the gambling industry and its representatives

| **Protect policy** | **% (95% CI)** |
| --- | --- |
| Strongly support | 56.4 (55.5, 57.3) |
| Tend to support | 19.2 (18.5, 20.0) |
| Neither support nor oppose | 13.0 (12.4, 13.6) |
| Tend to oppose | 2.2 (1.9, 2.5) |
| Strongly oppose | 1.8 (1.5, 2.0) |
| Don’t know | 7.4 (6.9, 7.9) |

Table 39: How strongly, if at all, would you support the following measure…? Requiring the gambling industry to pay a levy to government for measures to reduce and prevent harm from gambling

| **Levy** | **% (95% CI)** |
| --- | --- |
| Strongly support | 50.7 (49.8, 51.6) |
| Tend to support | 23.5 (22.7, 24.3) |
| Neither support nor oppose | 13.3 (12.7, 13.9) |
| Tend to oppose | 3.5 (3.2, 3.9) |
| Strongly oppose | 2.8 (2.5, 3.1) |
| Don’t know | 6.2 (5.7, 6.6) |

Table 40: Currently, the legal age for gambling varies according to the activity, with some gambling activity having no minimum legal age (e.g. gambling in video games). How strongly, if at all, would you support or oppose the following? Setting a minimum legal age of 18 for all forms of gambling

| **Minimum gambling age** | **% (95% CI)** |
| --- | --- |
| Strongly support | 67.4 (66.6, 68.3) |
| Tend to support | 20.0 (19.3, 20.7) |
| Neither support nor oppose | 6.7 (6.3, 7.2) |
| Tend to oppose | 1.4 (1.2, 1.7) |
| Strongly oppose | 0.9 (0.7, 1.1) |
| Don’t know | 3.5 (3.1, 3.8) |

Table 41: How strongly, if at all, would you support or oppose the following measure…? Complete ban on all gambling advertising

| **Advertising ban** | **% (95% CI)** |
| --- | --- |
| Strongly support | 43.6 (42.7, 44.5) |
| Tend to support | 19.0 (18.3, 19.7) |
| Neither support nor oppose | 18.2 (17.5, 18.9) |
| Tend to oppose | 8.7 (8.2, 9.2) |
| Strongly oppose | 5.2 (4.8, 5.6) |
| Don’t know | 5.3 (4.9, 5.8) |

Table 42: How strongly, if at all, would you support or oppose the following measure…? Ban on advertising gambling on broadcast media, including TV and radio

| **Broadcast advertising ban** | **% (95% CI)** |
| --- | --- |
| Strongly support | 48.2 (47.3, 49.1) |
| Tend to support | 21.7 (21.0, 22.5) |
| Neither support nor oppose | 16.0 (15.3. 16.6) |
| Tend to oppose | 5.7 (5.3, 6.2) |
| Strongly oppose | 3.2 (2.8, 3.5) |
| Don’t know | 5.2 (4.7, 5.6) |

Table 43: How strongly, if at all, would you support or oppose the following measure…? Not allowing gambling advertising on social media and online

| **Online advertising** | **% (95% CI)** |
| --- | --- |
| Strongly support | 49.0 (48.1, 50.0) |
| Tend to support | 21.7 (21.0, 22.5) |
| Neither support nor oppose | 15.6 (14.9, 16.2) |
| Tend to oppose | 5.0 (4.6, 5.4) |
| Strongly oppose | 3.3 (3.0, 3.6) |
| Don’t know | 5.4 (4.9, 5.8) |

Table 44: How strongly, if at all, would you support or oppose the following measure…? Stopping gambling adverts from being shown on TV and radio before 9pm

| **Watershed advertising ban** | **% (95% CI)** |
| --- | --- |
| Strongly support | 54.1 (53.2, 55.0) |
| Tend to support | 22.8 (22.1, 23.6) |
| Neither support nor oppose | 12.3 (11.7, 12.9) |
| Tend to oppose | 3.2 (2.9, 3.5) |
| Strongly oppose | 2.6 (2.3, 2.9) |
| Don’t know | 5.0 (4.6, 5.4) |

Table 45: How strongly, if at all, would you support or oppose the following measure…? Not allowing gambling companies to sponsor sporting events or teams

| **Sports sponsorship** | **% (95% CI)** |
| --- | --- |
| Strongly support | 45.9 (45.0, 46.9) |
| Tend to support | 19.7 (18.9, 20.4) |
| Neither support nor oppose | 17.7 (17.0, 18.4) |
| Tend to oppose | 7.0 (6.5, 7.4) |
| Strongly oppose | 4.2 (3.9, 4.6) |
| Don’t know | 5.5 (5.1, 6.0) |

Table 46: How strongly, if at all, would you support or oppose the following measure…? Only allowing gambling advertising in cinemas for films with an 18 certificate

| **Sports sponsorship** | **% (95% CI)** |
| --- | --- |
| Strongly support | 47.0 (46.1, 47.9) |
| Tend to support | 26.4 (25.6, 27.2) |
| Neither support nor oppose | 14.0 (13.4, 14.7) |
| Tend to oppose | 3.2 (2.8, 3.5) |
| Strongly oppose | 3.7 (3.4, 4.1) |
| Don’t know | 5.8 (5.3, 6.2) |

Table 47: How strongly, if at all, would you support or oppose the following measure…? Not allowing gambling companies to sponsor music and cultural events

| **Cultural events sponsorship** | **% (95% CI)** |
| --- | --- |
| Strongly support | 46.1 (45.2, 47.0) |
| Tend to support | 20.6 (19.9, 21.4) |
| Neither support nor oppose | 17.7 (17.0, 18.4) |
| Tend to oppose | 6.2 (5.8, 6.7) |
| Strongly oppose | 3.8 (3.4, 4.1) |
| Don’t know | 5.5 (5.1, 5.9) |

Table 48: How strongly, if at all, would you support or oppose the following measure…? Not allowing gambling advertising in outdoor and public spaces such as streets, parks and public transport

| **Public spaces advertising** | **% (95% CI)** |
| --- | --- |
| Strongly support | 47.6 (46.7, 48.5) |
| Tend to support | 23.3 (22.5, 24.1) |
| Neither support nor oppose | 16.3 (15.6, 16.9) |
| Tend to oppose | 4.8 (4.4, 5.2) |
| Strongly oppose | 2.8 (2.5, 3.1) |
| Don’t know | 5.3 (4.9, 5.7) |

Table 49: How strongly, if at all, would you support or oppose the following measure…? Including a health warning on all gambling advertising to communicate the risks associated with gambling

| **Health warnings** | **% (95% CI)** |
| --- | --- |
| Strongly support | 49.1 (48.1, 50.0) |
| Tend to support | 26.6 (25.8, 27.4) |
| Neither support nor oppose | 14.0 (13.3, 14.6) |
| Tend to oppose | 2.7 (2.4, 3.0) |
| Strongly oppose | 2.4 (2.1, 2.7) |
| Don’t know | 5.2 (4.8, 5.6) |

**Table 5**: Overall support and adjusted odds ratios^1^ for support of gambling policies (weighted, N_unweighted_=12,271)

| **Policy** | **Overall support (%)** | **Age (ref: 18-24)** | | | | | **Sex (ref: men)** | **Social grade (ref: ABC1)** | **Currently smoking (ref: no)** | **BMI ≥30 (ref: no)** | **Drinking alcohol above guidelines (ref: no)** | **Gambling daily (ref: no)** |
| --- | --- | --- | --- | --- | --- | --- | --- | --- | --- | --- | --- | --- |
|  |  | 25-34 | 35-44 | 45-54 | 55-64 | 65+ | Women | C2DE | Yes | Yes | Yes | Yes |
| Health policy should be protected from the influence of the gambling industry | 75.7 | 1.05 (1.01, 1.08) | 1.07 (1.03, 1.10) | 1.11 (1.08, 1,15) | 1.15 (1.11, 1.18) | 1.20 (1.16, 1.23) | 0.99 (0.97, 1.00) | 0.92 (0.90, 0.93) | 0.89 (0.87, 0.92) | 0.99 (0.97, 1.00) | 0.96 (0.94, 0.99) | 0.95 (0.88, 1.03) |
| Levy on gambling industry to reduce and prevent harm from gambling | 74.2 | 1.04 (1.01, 1.08) | 1.06 (1.03, 1.10) | 1.12 (1.08, 1.08) | 1.14 (1.10, 1.10) | 1.19 (1.15, 1.15) | 1.01 (1.00, 1.03) | 0.91 (0.90, 0.93) | 0.89 (0.87, 0.92) | 0.98 (0.96, 1.00) | 0.95 (0.93, 0.97) | 0.98 (0.90, 1.06) |
| Setting a minimum age of 18 for all forms of gambling | 87.4 | 1.04 (1.01, 1.07) | 1.06 (1.03, 1.09) | 1.12 (1.09, 1.15) | 1.14 (1.11, 1.11) | 1.17 (1.14, 1.14) | 1.03 (1.02, 1.05) | 0.94 (0.93, 0.96) | 0.95 (0.93, 0.97) | 0.99 (0.98, 1.01) | 0.96 (0.94, 0.98) | 0.99 (0.93, 1.05) |
| Complete ban on all gambling advertising | 62.6 | 1.03 (0.99, 1.07) | 1.04 (1.00, 1.08) | 1.10 (1.06, 1.14) | 1.12 (1.08, 1.16) | 1.19 (1.16, 1.23) | 1.08 (1.06, 1.10) | 0.95 (0.93, 0.96) | 0.92 (0.89, 0.94) | 0.99 (0.97, 1.01) | 0.92 (0.90, 0.95) | 0.85 (0.79, 0.93) |
| Ban on advertising gambling on broadcast media, including TV and radio | 70.0 | 1.03 (0.99, 1.06) | 1.05 (1.02, 1.09) | 1.09 (1.05, 1.13) | 1.14 (1.10, 1.18) | 1.23 (1.10, 1.27) | 1.06 (1.04, 1.07) | 0.94 (0.92, 0.95) | 0.91 (0.89, 0.94) | 0.99 (0.97, 1.01) | 0.93 (0.91, 0.95) | 0.84 (0.77, 0.91) |
| Not allowing gambling advertising on social media and online | 70.8 | 1.03 (1.00, 1.07) | 1.05 (1.02, 1.09) | 1.10 (1.07, 1.14) | 1.15 (1.11, 1.19) | 1.21 (1.17, 1.25) | 1.07 (1.05, 1.09) | 0.94 (0.92, 0.95) | 0.90 (0.88, 0.93) | 0.99 (0.97, 1.01) | 0.93 (0.91, 0.95) | 0.86 (0.79, 0.93) |
| Stopping gambling adverts from being shown on TV and radio before 9pm | 76.9 | 1.02 (0.99, 1.06) | 1.06 (1.02, 1.09) | 1.12 (1.08, 1.16) | 1.16 (1.13, 1.20) | 1.22 (1.19, 1.26) | 1.06 (1.04, 1.07) | 0.94 (0.93, 0.96) | 0.90 (0.87, 0.92) | 0.99 (0.97, 1.00) | 0.93 (0.91, 0.95) | 0.89 (0.82, 0.97) |
| Not allowing gambling companies to sponsor sporting events or teams | 65.6 | 1.01 (0.98, 1.05) | 1.03 (0.99, 1.06) | 1.06 (1.02, 1.10) | 1.09 (1.05, 1.12) | 1.13 (1.10, 1.17) | 1.06 (1.04, 1.08) | 0.93 (0.91, 0.95) | 0.89 (0.86, 0.91) | 0.99 (0.97, 1.01) | 0.95 (0.93, 0.97) | 0.84 (0.78, 0.92) |
| Only allowing gambling advertising in cinemas for films with an 18 certificate | 73.3 | 1.02 (0.99, 1.06) | 1.06 (1.02, 1.09) | 1.12 (1.08, 1.16) | 1.16 (1.13, 1.20) | 1.22 (1.19, 1.26) | 1.06 (1.04, 1.07) | 0.94 (0.93, 0.96) | 0.90 (0.87, 0.92) | 0.99 (0.97, 1.00) | 0.93 (0.91, 0.95) | 0.89 (0.82, 0.97) |
| Not allowing gambling companies to sponsor music and cultural events | 66.7 | 1.04 (1.00, 1.07) | 1.05 (1.01, 1.09) | 1.08 (1.04, 1.11) | 1.09 (1.06, 1.13) | 1.14 (1.11, 1.18) | 1.06 (1.04, 1.08) | 0.93 (0.92, 0.95) | 0.90 (0.87, 0.92) | 0.99 (0.97, 1.01) | 0.93 (0.91, 0.96) | 0.85 (0.78, 0.92) |
| Ban on gambling advertising in outdoor and public spaces such as streets, parts and public transport | 70.9 | 1.02 (0.99, 1.06) | 1.04 (1.01, 1.08) | 1.07 (1.03, 1.11) | 1.11 (1.08, 1.15) | 1.17 (1.13, 1.20) | 1.06 (1.04, 1.08) | 0.94 (0.93, 0.96) | 0.89 (0.86, 0.91) | 0.98 (0.96, 1.00) | 0.94 (0.92, 0.96) | 0.87 (0.80, 0.95) |
| Including a health warning on all gambling advertising | 75.7 | 1.02 (0.99, 1.06) | 1.02 (0.99, 1.05) | 1.07 (1.04, 1.11) | 1.10 (1.07, 1.14) | 1.13 (1.09, 1.16) | 1.03 (1.02, 1.05) | 0.93 (0.92, 0.95) | 0.89 (0.81, 0.92) | 1.00 (0.98, 1.02) | 0.94 (0.92, 0.96) | 0.88 (0.81, 0.95) |

^1^Respective other variables were used for adjustment
