# Supplementary material for: Public opinion on policy interventions for regulating four unhealthy commodity industries: a cross-sectional online survey of a representative sample of British adults 2023
Source: BMC Public Health. 2025 Nov 21;25:4402. doi: 10.1186/s12889-025-25271-x (PMC12754964; doi:10.1186/s12889-025-25271-x)
Supplement: Supplementary file 2 — Additional file 2. STROBE checklist. [file 12889_2025_25271_MOESM2_ESM.docx]

**STROBE Checklist for the Present Study**

Checklist of items that should be included in reports of ***cross-sectional studies***

|  | Item No | Recommendation | Present Study (Y/N and justification) |
| --- | --- | --- | --- |
| **Title and abstract** | 1 | (*a*) Indicate the study’s design with a commonly used term in the title or the abstract | **Y** (Abstract - design) |
|  |  | (*b*) Provide in the abstract an informative and balanced summary of what was done and what was found | **Y** (Abstract – design, participants, measures, analysis, main findings) |
| Introduction | | |  |
| Background/rationale | 2 | Explain the scientific background and rationale for the investigation being reported | **Y** (Introduction – background and rationale provided) |
| Objectives | 3 | State specific objectives, including any prespecified hypotheses | **Y** (Introduction – research questions stated) |
| Methods | | |  |
| Study design | 4 | Present key elements of study design early in the paper | **Y** (Methods – cross-sectional survey design - sample, recruitment, outcome measures and analysis methods clearly stated) |
| Setting | 5 | Describe the setting, locations, and relevant dates, including periods of recruitment, exposure, follow-up, and data collection | **Y** (Methods – secondary analysis of the ASH Smokefree GB annual survey from 2023, sampling methods by YouGov described for participant recruitment) |
| Participants | 6 | (*a*) Give the eligibility criteria, and the sources and methods of selection of participants | **Y** (Sample and recruitment – YouGov’s active sampling method from their panel described) |
| Variables | 7 | Clearly define all outcomes, exposures, predictors, potential confounders, and effect modifiers. Give diagnostic criteria, if applicable | **Y** (Methods – outcome measures around support for policy measures, harmful consumption of unhealthy commodities and sociodemographic characteristics clearly stated; variables around sociodemographic characteristics and harmful consumption of unhealthy commodities were adjusted for in the logistic regression). |
| Data sources/ measurement | 8* | For each variable of interest, give sources of data and details of methods of assessment (measurement). Describe comparability of assessment methods if there is more than one group | **Y** (Methods – outcome measures around sociodemographic characteristics were derived from YouGov’s existing panel; consumption of unhealthy commodities measured in the survey through self-report, with obesity used as a proxy of exposure to unhealthy food and drink, support for policy measures directly questioned through the survey). |
| Bias | 9 | Describe any efforts to address potential sources of bias | **Y** (Analysis – all data were weighted by age, sex, region, socio-economic position and ethnicity to be representative of GB adults; logistic regression was used to control for covariates when examining associations between support for policies, sociodemographic characteristics and harmful consumption of unhealthy commodities). |
| Study size | 10 | Explain how the study size was arrived at | **N – (**secondary analysis of the annual ASH Smokefree Survey, 12,271 adults were invited to participate from YouGov’s existing panel) |
| Quantitative variables | 11 | Explain how quantitative variables were handled in the analyses. If applicable, describe which groupings were chosen and why | **Y** – (Analysis – for the first research question the degree of public support for different policies were plotted graphically; for the second research question, responses were dichotomised and adjusted odds ratios calculated using logistic regression methods) |
| Statistical methods | 12 | (*a*) Describe all statistical methods, including those used to control for confounding | **Y** (as for 11. – logistic regression methods were used to control for covariates when examining associations between support for policies, sociodemographic characteristics and harmful consumption of unhealthy commodities). |
|  |  | (*b*) Describe any methods used to examine subgroups and interactions | n/a |
|  |  | (*c*) Explain how missing data were addressed | **Y** (Methods – analysis – missing values were imputed using multivariate imputation by chained equations). |
|  |  | (*d*) If applicable, describe analytical methods taking account of sampling strategy | n/a |
|  |  | (*e*) Describe any sensitivity analyses | n/a |
| Results | | |  |
| Participants | 13* | (a) Report numbers of individuals at each stage of study—eg numbers potentially eligible, examined for eligibility, confirmed eligible, included in the study, completing follow-up, and analysed | n/a – cross sectional survey design – secondary analysis of an existing survey. |
|  |  | (b) Give reasons for non-participation at each stage | **Y** – (Results – sample characteristics – missing variables clearly stated and available in supplementary material) |
|  |  | (c) Consider use of a flow diagram | n/a |
| Descriptive data | 14* | (a) Give characteristics of study participants (eg demographic, clinical, social) and information on exposures and potential confounders | **Y** – (Results – tables 1, 2,3,4 and 5). |
|  |  | (b) Indicate number of participants with missing data for each variable of interest | **Y** – (the number and percent of missingness for each variable is reported in the supplementary material). |
| Outcome data | 15* | Report numbers of outcome events or summary measures | **Y** – (Results – Sample characteristics in Table 1; for research question 1, percentage support for policy measures shown in Figures 1-3; for research question 2, overall support for policy areas and adjusted odds ratios for sociodemographic characteristics and harmful consumption of unhealthy commodities shown in Tables 2-5). |
| Main results | 16 | (*a*) Give unadjusted estimates and, if applicable, confounder-adjusted estimates and their precision (eg, 95% confidence interval). Make clear which confounders were adjusted for and why they were included | **Y** – (Results – For research question 1, percentage support for policy measures shown in Figures 1-3; for research question 2, overall support for policy areas and adjusted odds ratios for sociodemographic characteristics and harmful consumption of unhealthy commodities shown in Tables 2-5) |
|  |  | (*b*) Report category boundaries when continuous variables were categorized | **Y** – (Results – sample characteristics, table 1). |
|  |  | (*c*) If relevant, consider translating estimates of relative risk into absolute risk for a meaningful time period | n/a |
| Other analyses | 17 | Report other analyses done—eg analyses of subgroups and interactions, and sensitivity analyses | n/a |
| Discussion | | |  |
| Key results | 18 | Summarise key results with reference to study objectives | **Y** – (Discussion – summary of key findings) |
| Limitations | 19 | Discuss limitations of the study, taking into account sources of potential bias or imprecision. Discuss both direction and magnitude of any potential bias | **Y** – (Discussion – strengths and limitations of the study). |
| Interpretation | 20 | Give a cautious overall interpretation of results considering objectives, limitations, multiplicity of analyses, results from similar studies, and other relevant evidence | **Y** – (Discussion – comparison with previous research and interpretation of findings; implications of findings; conclusions). |
| Generalisability | 21 | Discuss the generalisability (external validity) of the study results | **Y** – (Discussion – strengths and limitations of the study). |
| Other information | | |  |
| Funding | 22 | Give the source of funding and the role of the funders for the present study and, if applicable, for the original study on which the present article is based | **Y** (explicitly stated at the end of the paper). |

*Give information separately for exposed and unexposed groups.
